# Case Illustration of the Natural History of Left Dominant Arrhythmogenic Cardiomyopathy

**DOI:** 10.31486/toj.23.0057

**Published:** 2024

**Authors:** Corry B. Sanford, Jerry Fan, Yinan Hua, Lazaros Nikolaidis, Whitney Edmister, Sarah Payne, Hari Dandapantula, Manik Veer, Vinh Nguyen

**Affiliations:** ^1^ Department of Internal Medicine, Baylor Scott & White Medical Center, Temple, TX; ^2^ Division of Cardiology, Baylor Scott & White Medical Center, Temple, TX; ^3^ Department of Pathology, Baylor Scott & White Medical Center, Temple, TX; ^4^ Department of Radiology, Baylor Scott & White Medical Center, Temple, TX; ^5^ Department of Cardiology, MercyOne Iowa Heart Center, Des Moines, IA

**Keywords:** *Arrhythmogenic left ventricular cardiomyopathy*, *cardiomyopathies*, *desmoplakins*, *recurrent myocarditis*

## Abstract

**Background:** Arrhythmogenic left ventricular cardiomyopathy is an increasingly recognized cause of recurrent myocarditis, a mimicker of acute coronary syndrome, and an important cause of malignant ventricular arrythmias and heart failure. Desmoplakin is a protein that is critical to maintaining the structural integrity of the myocardium. Disruption of desmoplakin leads to fibrofatty infiltration of the myocardium which leads to congestive heart failure, cardiac arrhythmias, and sudden cardiac death. However, desmoplakin cardiomyopathy is often misdiagnosed, resulting in significant morbidity and mortality. We report 2 contrasting cases illustrating the natural history—hot and cold phases—of arrhythmogenic left ventricular cardiomyopathy.

**Case Series:** The first case demonstrates a common phenotypic presentation of desmoplakin cardiomyopathy manifested as recurrent myocarditis and myocardial injury representing the hot phase. The second case is an undulating course of chronic systolic heart failure and ventricular arrhythmias representing the cold phase.

**Conclusion:** Arrhythmogenic cardiomyopathy manifests as a spectrum of disease processes that involve the right, left, or both ventricles. Mutations in the desmoplakin gene are often associated with a left dominant ventricular cardiomyopathy. Diagnosis remains difficult as the condition has no signature clinical presentation, and imaging findings are variable.

## INTRODUCTION

Arrhythmogenic cardiomyopathy manifests as a spectrum of myocardial disorders characterized by progressive myocardial damage caused by fibrofatty replacement of the myocardium and resulting malignant ventricular arrhythmias.^1,2^ Arrhythmogenic cardiomyopathy can present with heart failure, arrhythmias, and sudden cardiac death.^2^ Previously believed to be isolated to right ventricular involvement, arrhythmogenic cardiomyopathy is now understood to involve the right, left, or both ventricles.^1^

Arrhythmogenic left ventricular cardiomyopathy is a rare, genetically inheritable disease associated with a desmoplakin mutation that alters the protein that determines the structural integrity of the myocardium.^1,2^ Clinical presentation is highly variable and often includes ventricular arrhythmias, myocardial injury with left ventricular dysfunction, or recurrent myocarditis.^1,3^ Patients are often misdiagnosed as having recurrent myocarditis because of alternating periods of the hot and cold phases of the disease. In the natural course of arrhythmogenic left ventricular cardiomyopathy, patients often cycle between the hot and cold phases, although not all patients will undergo both phases. Patients often seek medical attention during the hot phase because of symptoms of chest pain, while those in the cold phase often have a long asymptomatic course until an episode of decompensated heart failure.

We report 2 cases of arrhythmogenic left ventricular cardiomyopathy secondary to desmoplakin mutation. The patient in the first case had recurrent hot phases demonstrated by recurrent myocarditis. The patient in the second case had an indolent subclinical cold phase characterized by development of a dilated cardiomyopathy.

## CASE SERIES

### Case 1

A 43-year-old Hispanic female presented with acute substernal chest pain. Workup revealed elevated troponin I (0.59 ng/mL; reference range, 0.00-0.09 ng/mL), white blood cell count (7.9 × 10^9^/L; reference range, 4.8-10.8 × 10^9^/L), C-reactive protein (1.2 mg/L; reference range, 0.0-3.2 mg/L), sedimentation rate (13 mmol/hr; reference range, 0-20 mmol/hr), and ST depressions in the inferior leads on electrocardiogram (ECG). Transthoracic echocardiogram demonstrated a normal left ventricular ejection fraction (LVEF) (60%-65%; reference range [female], 54%-74%). Coronary angiogram did not show significant epicardial coronary disease. The patient presented 3 years later with similar acute substernal chest pain associated with elevated troponin I (3.06 ng/mL; reference range, 0.00-0.09 ng/mL).

**Figure 1.**
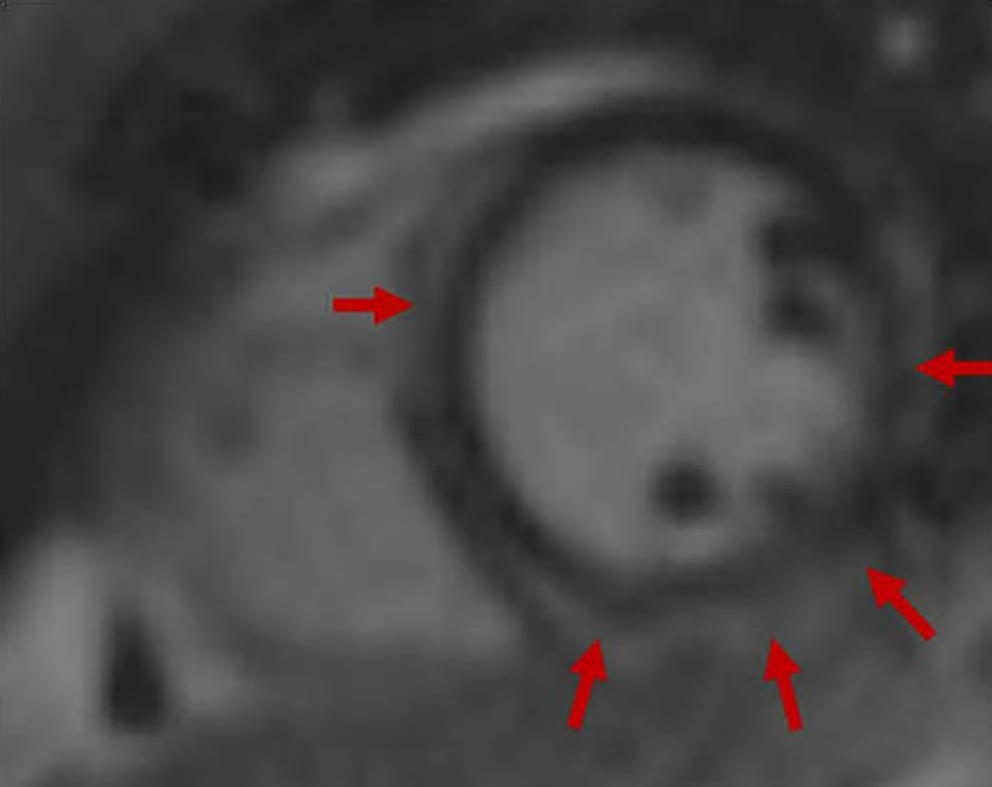
Case 1: Cardiac magnetic resonance imaging shows a ring-like pattern (arrows) of late gadolinium enhancement with near circumferential involvement of the mesocardium and epicardium.

**Figure 2.**
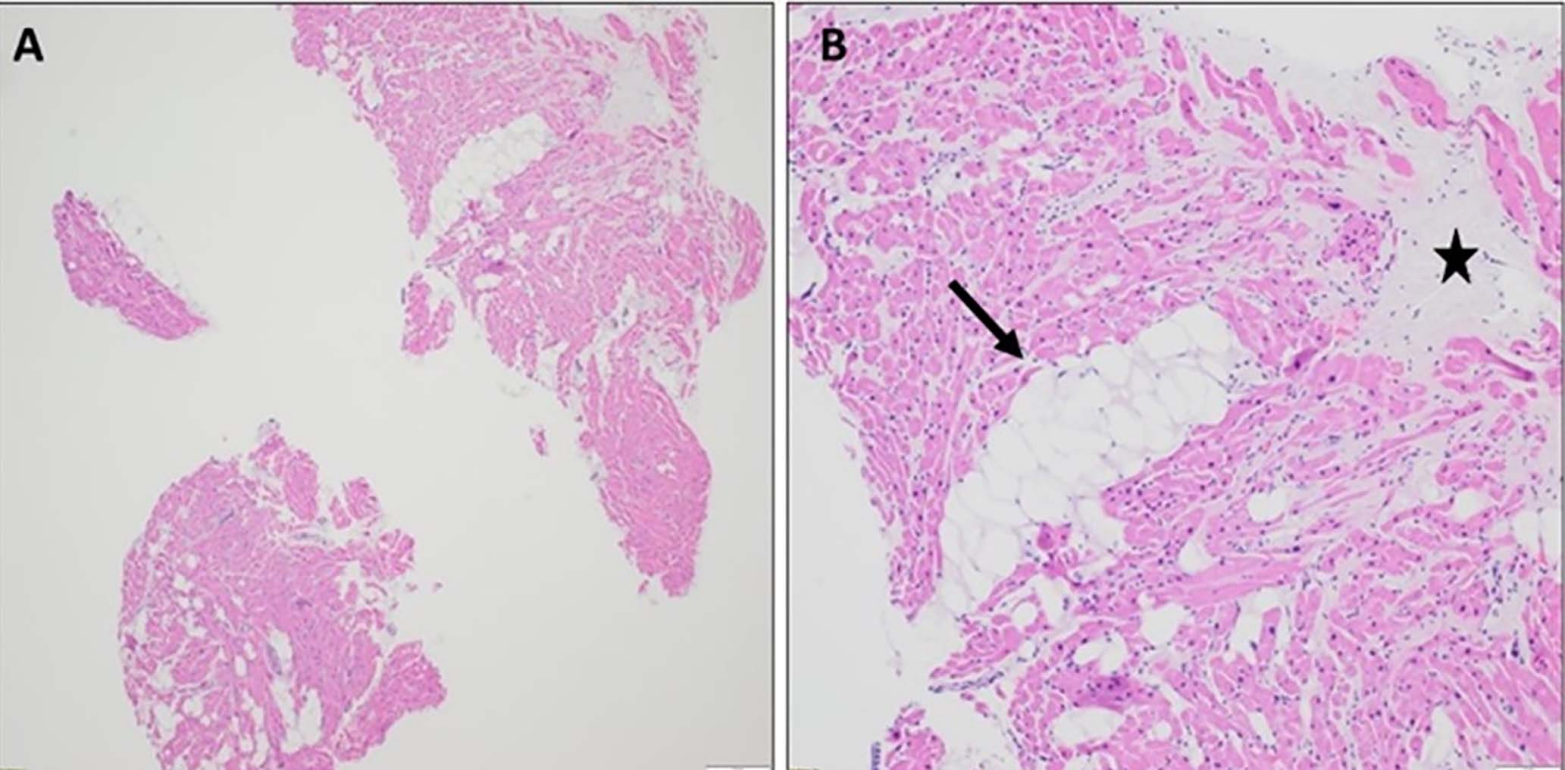
Case 1: Myocardium shows pathologic infiltration of adipocytes (arrows) and intestinal fibrosis (star) at (A) 40× magnification and (B) 100× magnification.

Echocardiogram and coronary angiogram remained grossly unchanged. Cardiac magnetic resonance imaging (MRI) was notable for a ring-like pattern of late gadolinium enhancement showing near circumferential involvement of the mesocardium and epicardium, concerning for arrhythmogenic left ventricular cardiomyopathy (Figure 1). Right ventricular septal endomyocardial biopsy was notable for pathologic adipocyte infiltrate and interstitial fibrosis without evidence of acute myocardial injury, vasculitis, or amyloidosis (Figure 2).

The patient subsequently underwent genetic testing that revealed a heterozygous pathogenic variant of desmoplakin, c.4003C>T (p.Gln1335*). Electrophysiology study determined that the patient did not have inducible ventricular tachycardia; therefore, implantable cardioverter defibrillator placement was initially deferred. However, after discussion with the geneticist and because of a family history of unexplained sudden cardiac death (father), the patient decided to undergo placement of a subcutaneous implantable cardioverter defibrillator for primary prevention. She was started on a prednisone taper (40 mg twice daily, then once daily with an incremental 10 mg decreasing dose every week), colchicine (0.6 mg twice daily for 3 months, then colchicine 0.6 mg daily), and aspirin (325 mg daily). She established with the Advanced Heart Failure Clinic and genetics team and follows up every 2 to 3 months. Family genetic screening was performed on her 2 sons who both tested negative for her particular desmoplakin gene mutation; however, 1 of her sons was diagnosed with Carvajal syndrome, a variant of the desmoplakin gene mutation.

### Case 2

**Figure 3.**
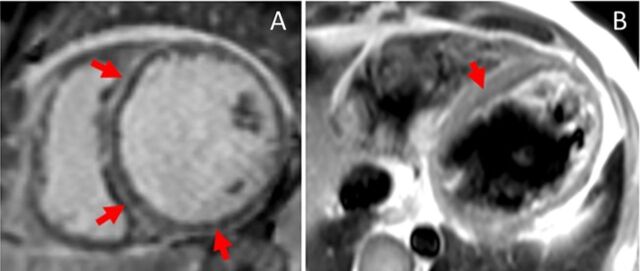
Case 2: Cardiac magnetic resonance imaging shows (A) contiguous mesocardial and epicardial late gadolinium enhancement in a ring-like distribution (arrows), and (B) matched hyperintensity on T2-weighted double inversion recovery, indicating fat infiltration.

**Figure 4.**
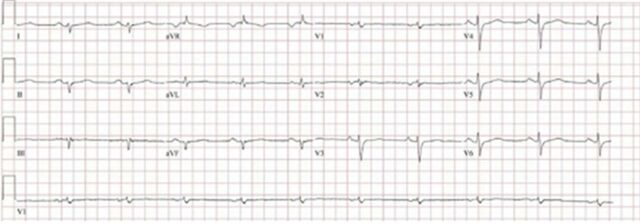
**Case 2: Electrocardiogram with notable low limb voltage, fulfilling the depolarization abnormality of the Padua criteria.** ^4^

A 46-year-old Hispanic male with chronic systolic heart failure (LVEF 20%; reference range [male], 52%-72%) presented with left-sided facial droop attributable to a right middle cerebral artery ischemic infarct. Transthoracic echocardiogram demonstrated a severely dilated left ventricle with severely depressed left ventricular systolic function and a left apical thrombus. Cardiac MRI revealed a ring-like pattern of late gadolinium enhancement showing near circumferential involvement of the mesocardium and epicardium (Figure 3). Coronary angiogram was negative for significant epicardial coronary disease. ECG demonstrated low limb voltage (Figure 4).

Genetic testing revealed a heterozygous pathogenic variant of desmoplakin, c.5680_5683del (p.Ser1894Leufs*34). The patient met multiple Padua criteria for arrhythmogenic left ventricular cardiomyopathy: morphofunctional abnormalities, structural myocardial abnormalities, depolarization abnormalities (low limb voltage), ventricular arrhythmias, and a pathologic variant known to cause arrhythmogenic cardiomyopathy.^4^

The patient was placed on guideline-directed medical therapy for systolic heart failure: empagliflozin (10 mg daily), metoprolol succinate (12.5 mg daily), sacubitril-valsartan (12-13 mg twice daily), spironolactone (12.5 mg daily), and torsemide (20 mg daily), as well as apixaban (5 mg twice daily) for left ventricular thrombus. The patient developed sustained ventricular arrhythmias and underwent implantable cardioverter defibrillator placement. Follow-up transthoracic echocardiogram 4 months later demonstrated no significant myocardial recovery with LVEF 20%-25%. The patient established with the Advanced Heart Failure Clinic and follows up every 2 to 3 months. He was counseled about genetic testing for future offspring.

## DISCUSSION

Arrhythmogenic cardiomyopathy remains a relatively rare disease with an estimated prevalence of 1 in 2,000 to 5,000 individuals, but this prevalence may be underestimated because of incomplete penetrance; the relationship between genotypic and phenotypic expression is still poorly understood, and mechanisms are still being elucidated.^5^ The diagnosis of arrhythmogenic left ventricular cardiomyopathy remains challenging as the disease can mimic other cardiac pathologies and lacks sufficient diagnostic criteria.^6^ In a group of individuals with endomyocardial biopsy–proven arrhythmogenic left ventricular cardiomyopathy, application of the 2010 International Task Force Criteria for arrhythmogenic right ventricular cardiomyopathy resulted in <50% of individuals meeting the criteria for diagnosis.^5-7^ As such, the 2020 Padua criteria proposed new diagnostic criteria for left-sided arrhythmogenic cardiomyopathy, introducing tissue characterization via late gadolinium enhancement on cardiac MRI, ECG depolarization/repolarization abnormalities, and ventricular arrhythmias specific for left-sided involvement.^4^

Desmosomes serve as intercellular adhesive junctions between cardiomyocytes that provide cellular structural integrity.^1^ Mutations in desmosomes are implicated in approximately 50% of arrhythmogenic cardiomyopathy because of the loss of electromechanical coupling and subsequent lymphocyte infiltration and necrosis that lead to fibrofatty replacement of myocardium, arrhythmogenic potential, and left ventricular dysfunction.^1,3,5,7^ Mutations include genes that encode components of desmosomes such as plakoglobin, desmoplakin, plakophilin-2, desmoglein-2, and desmocollin-2.^8^ Genes encoding nondesmosomal proteins such as ion channels or cytoskeleton-associated proteins (such as desmin, lamin, and myosin heavy and light chains) are also implicated in arrhythmogenic cardiomyopathy.^8^ Patients have a structurally normal heart at birth, and penetrance occurs later in life. Phenotypic expression of desmoplakin mutations favors involvement of the left ventricle.^1,3,7^

Inflammation has been implicated in the development of myocardial damage caused by desmoplakin mutations, leading to phenotypic expression of arrhythmogenic left ventricular cardiomyopathy.^1^ Desmoplakin cardiomyopathy follows a distinctive natural history with episodic myocardial injury resulting in myocardial fibrosis, dysfunction, and eventual replacement with fibrofatty tissue.^1,7^ Cold phases result in subclinical but continuous cell death and alternate with hot phases that result in episodes that mimic acute coronary syndrome or recurrent myocarditis.^1,3^

The clinical manifestations of genetic cardiomyopathy associated with recurrent episodes of acute myocarditis have been reported in multiple case series, suggesting that myocarditis may be an early phenotypic manifestation of genetic cardiomyopathy with worse clinical outcomes compared to cases of nongenetic cardiomyopathy.^1,3,5^ Although arrhythmogenic cardiomyopathy may present clinically as myocarditis, late gadolinium enhancement may be distinctly different from the typical multifocal distribution and have a ring-like enhancement as demonstrated in our patients. Chen et al reported on a cohort of patients with dilated cardiomyopathy who did not show a significant correlation between late gadolinium enhancement pattern and left ventricular function.^9^ The Chen et al study suggests that patients with no or focal late gadolinium enhancement had less burden of ventricular tachyarrhythmias compared to patients with ring-like or multifocal late gadolinium enhancement. In contrast, no significant association was seen between the amount of late gadolinium enhancement and left ventricular function.^9^ Based on a study by Augusto et al demonstrating a genetic correlation, the ring pattern is considered a defining pattern in arrhythmogenic left ventricular cardiomyopathy.^10^

Our knowledge of arrhythmogenic cardiomyopathy has been substantially enhanced by the Padua criteria^4^ and by cardiac MRI tissue characterization. Due to our enhanced understanding of the natural history of arrhythmogenic cardiomyopathy, patients who were previously characterized as having idiopathic myocarditis or undifferentiated dilated cardiomyopathy are now provided a more individualized approach using biomarkers (troponins, brain natriuretic peptide, erythrocyte sedimentation rate, C-reactive protein) to predict sudden cardiac death. The extent and recurrence of myocardial injury from recurrent myocarditis is associated with worsening heart failure and sustained ventricular arrhythmias.^7^

## CONCLUSION

Arrhythmogenic left ventricular cardiomyopathy has a wide range of phenotypic expressions, including recurrent myocarditis and silent myocardial injury. A high level of suspicion is required for expedient diagnosis and treatment. Patients who present with recurrent unexplained myocarditis (no prior viral infection, medications, immunologic causes, or toxins) or dilated cardiomyopathy with no epicardial coronary artery disease should prompt further imaging studies (MRI) and genetic testing. Arrhythmogenic left ventricular cardiomyopathy is an underrecognized clinical entity often misdiagnosed as isolated myocarditis or undifferentiated dilated cardiomyopathy.

## Notes

*This article meets the Accreditation Council for Graduate Medical Education and the American Board of Medical Specialties Maintenance of Certification competencies for Patient Care and Medical Knowledge.*
